# Learning curve for biportal endoscopic posterior cervical foraminotomy determined using the cumulative summation test

**DOI:** 10.1186/s13018-023-03611-0

**Published:** 2023-02-27

**Authors:** Min-Seok Kang, Hyun-Jin Park, Sang-Min Park, Ki-Han You, Won-Jik Ju

**Affiliations:** 1 Department of Orthopedic Surgery, Korea University Anam Hospital, Korea University College of Medicine, Seoul, Republic of Korea; 2 Department of Orthopedic Surgery, Spine Center, Hallym University Kangnam Sacred Heart Hospital, Hallym University College of Medicine, 1, Singil-ro, Yeongdeungpo-gu,, Seoul, 07441 Republic of Korea; 3 Department of Orthopedic Surgery, Seoul National University Bundang Hospital, Seoul National University College of Medicine, Seoul, Republic of Korea

**Keywords:** Biportal full-endoscopy, Cumulative summation test, Learning curve, Posterior cervical foraminotomy

## Abstract

**Background:**

Learning curves describe the rate of performance improvements corresponding to the surgeon’s caseload, followed by a plateau where limited further improvements are observed. This study aimed to determine the learning curve for biportal full-endoscopic posterior cervical foraminotomy (BE-PCF) for the unilateral cervical foraminal disc.

**Methods:**

The learning curve was evaluated using a learning curve cumulative summation test (LC-CUSUM). The goal for the operation time was set to 78 min, which is the mean operation time (mOT) of percutaneous full-endoscopic posterior cervical foraminotomy (PE-PCF) performed by a senior surgeon. Moreover, clinical outcomes and post-operative complications were compared between the early and late learning periods 1 year post-operatively.

**Results:**

This study enrolled the first 50 patients who underwent single-level BE-PCF, performed by a single surgeon. The LC-CUSUM signalled competency for surgery at the 20th operation, indicating that sufficient evidence was obtained to prove that the surgeon was competent. The mOT was 71.29 ± 11.69 min in BE-PCF, 71.84 ± 12.61 min in the early learning period, and 67.83 ± 10.31 min in the late learning period (*p* = 0.254). There was no statistical difference in clinical outcomes, visual analogue scale scores, and neck disability index between both periods (*p* > 0.05). Four complications were recorded throughout the whole period, with three in the early period and one in the late period (*p* = 0.285).

**Conclusion:**

Our study shows that BE-PCF has a learning curve of 20 caseloads to achieve 90% proficiency, and it significantly reduces the operation time based on the performance of a senior surgeon proficient in PE-PCF.

**Supplementary Information:**

The online version contains supplementary material available at 10.1186/s13018-023-03611-0.

## Introduction

The evolvement of spinal endoscopic platforms and techniques has enabled surgeons to perform spinal decompression under indirect visualisation with more limited surgical exposure, thereby moving the point of anatomical perspective into the body of patients. This improvement reduces inpatient care requirements, lowers surgical expenditures, reduces pain, and expedites functional recovery [1, 2]. However, spinal endoscopy requires surgical experience and training to improve the surgical performance of surgeons who are familiar with direct surgical visualisation using a surgical microscope. These improvements in surgical performance and increases in experience and training are referred to as the learning curve (LC) [3].

Micro-endoscopy, percutaneous uniportal full-endoscopy (PE), and biportal full-endoscopy (BE) are the most commonly utilised surgical techniques, of which the entire process under endoscopic visualisation from skin incision to closure is called full-endoscopy [1]. This technique relies on indirect visualisation, where an endoscopic lens is placed in proximity to the surgical field, and a working channel that communicates between the surgical field and outside space must be secured. PE comprises the coexistence of an endoscopic lens and a working channel inside one endoscopic platform, whereas BE has a view and working channel separated independently. In this study, we postulated that these morphological differences between these full-endoscopic techniques might influence the LC.

PE-PCF has been standardised as a representative surgical technique for cervical foraminal disc disease since the introduction of the full-endoscopic technique for posterior cervical foraminotomy (PCF) in the early 1990s [4–7]. However, BE-PCF still lacks clinical evidence; therefore, this study aimed to determine the LC for BE-PCF based on PE-PCF in unilateral cervical foraminal disc disease.

## Materials and methods

### Study design and patient population

The institutional review board of the university hospital approved the design (IRB approval number: 2022-04-012) and protocol of this retrospective study and waived the requirement for informed consent. We retrospectively reviewed the electrical medical records of 50 consecutive patients who had undergone BE-PCF from September 2018 to February 2021. These patients complained of posterior neck pain and radicular pain simultaneously, and underwent BE-PCF by a single orthopaedic surgeon (MS K) who was proficient in open microscopic PCF and had 1 year (49 cases) of experience with biportal endoscopic lumbar surgery but not with BE-PCF.

The inclusion criteria were as follows: (1) age between 18 and 80 years; (2) clinical manifestations and physical examinations consistent with single-level, unilateral cervical spondylotic radiculopathy, which was refractory to > 6 weeks of conservative treatment; and (3) lateral and foraminal cervical disc herniation or stenosis confirmed through magnetic resonance imaging (MRI). The exclusion criteria were as follows: (1) patients with cervical myelopathy with cord signal change upon MRI, segmental instability, and presence of hypoplasia of the lateral mass and cervical deformities; (2) patients with central localisation of the disc herniation or multi-level cervical spinal stenosis; (3) patients with prior surgery at the same level; and (4) patients who had not been followed up for > 1 year.

### Surgical procedure

BE-PCF was performed under general anaesthesia with the patient placed in a modified prone position on a radiolucent frame, alongside a slight reverse Trendelenburg inclination accommodating fluoroscopy. The patients were subsequently prepared and waterproof and draped in a sterile condition. BE-PCF was performed using a general arthroscopic surgical system (diameter, 4 mm; working length, 12.5 cm; and 0° or 30° field of view of an endoscope; ConMed Linvatec, Florida, USA). Moreover, continuous fluid irrigation was maintained intraoperatively using an automated pressure-controlled pump system (10 K® Fluid System, ConMed Linvatec, Florida, USA).

**Fig. 1:**
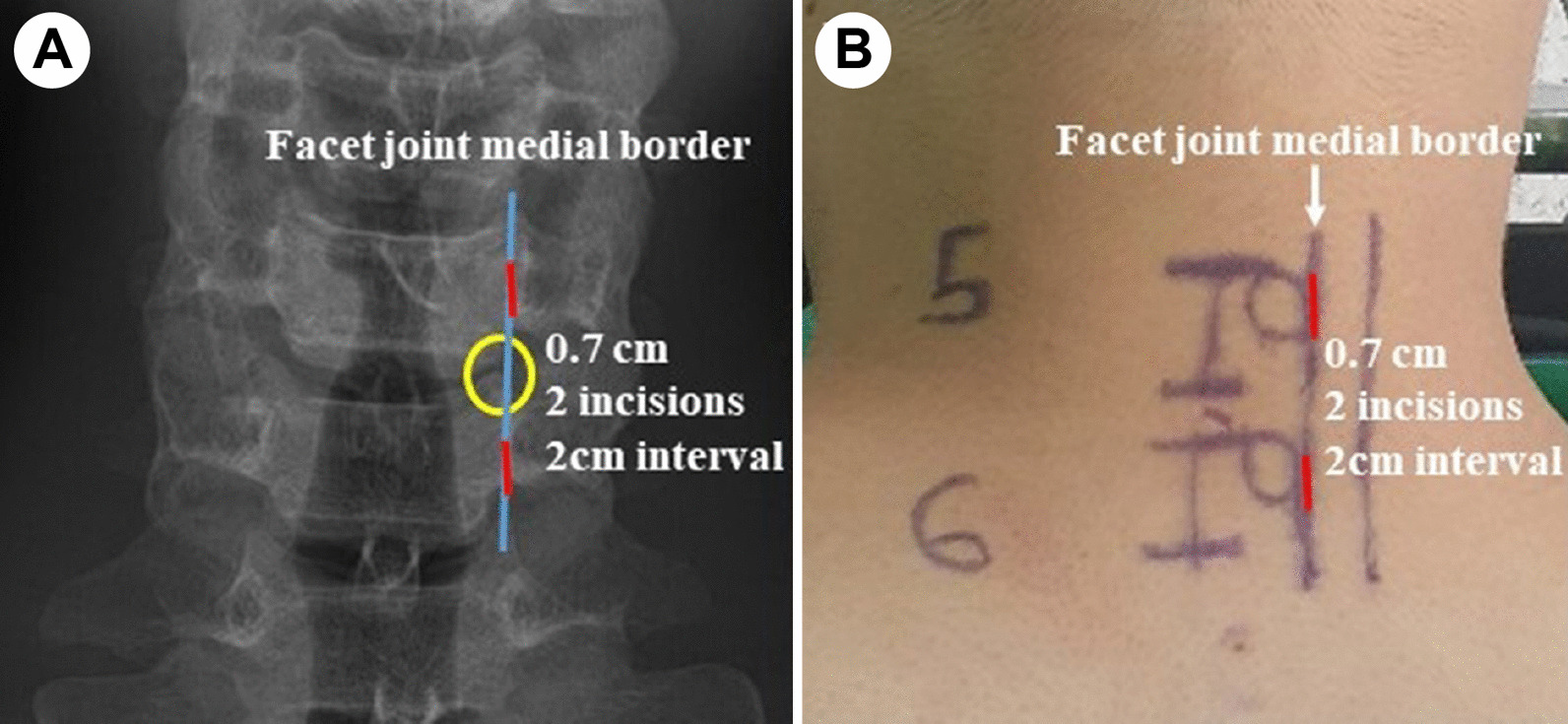
Skin incisions of biportal full-endoscopic posterior cervical foraminotomy (BE-PCF) (**A**, **B**). In BE-PCF, two skin incisions of 0.7 cm are made 2 cm apart around the “V”-point. Blue line, facet joint medial border; red line, incision site; and yellow circle, “V”-point

The target segment was confirmed under the guidance of C-arm fluoroscopy. Two 0.7 cm vertical skin incisions 2 cm apart were made around the “V”-point [6, 8], where the medial borders of the superior and inferior articular processes meet (Fig. 1). After performing muscle splitting using a sequential dilator onto the facet joint, a surgical endoscope was inserted, and proper endoscopic visualisation was subsequently obtained through tissue cauterisation using a bipolar radiofrequency ablator (Quantum 2, ArthroCare, CA, USA) and arthroscopic tissue shaver (Ergo Shaver handpiece; Conmed Linvatec; Largo, FL, USA). BE-PCF were performed in the same manner as conventional microscopic PCF, as described next, after obtaining adequate endoscopic visualisation.

**Fig. 2:**
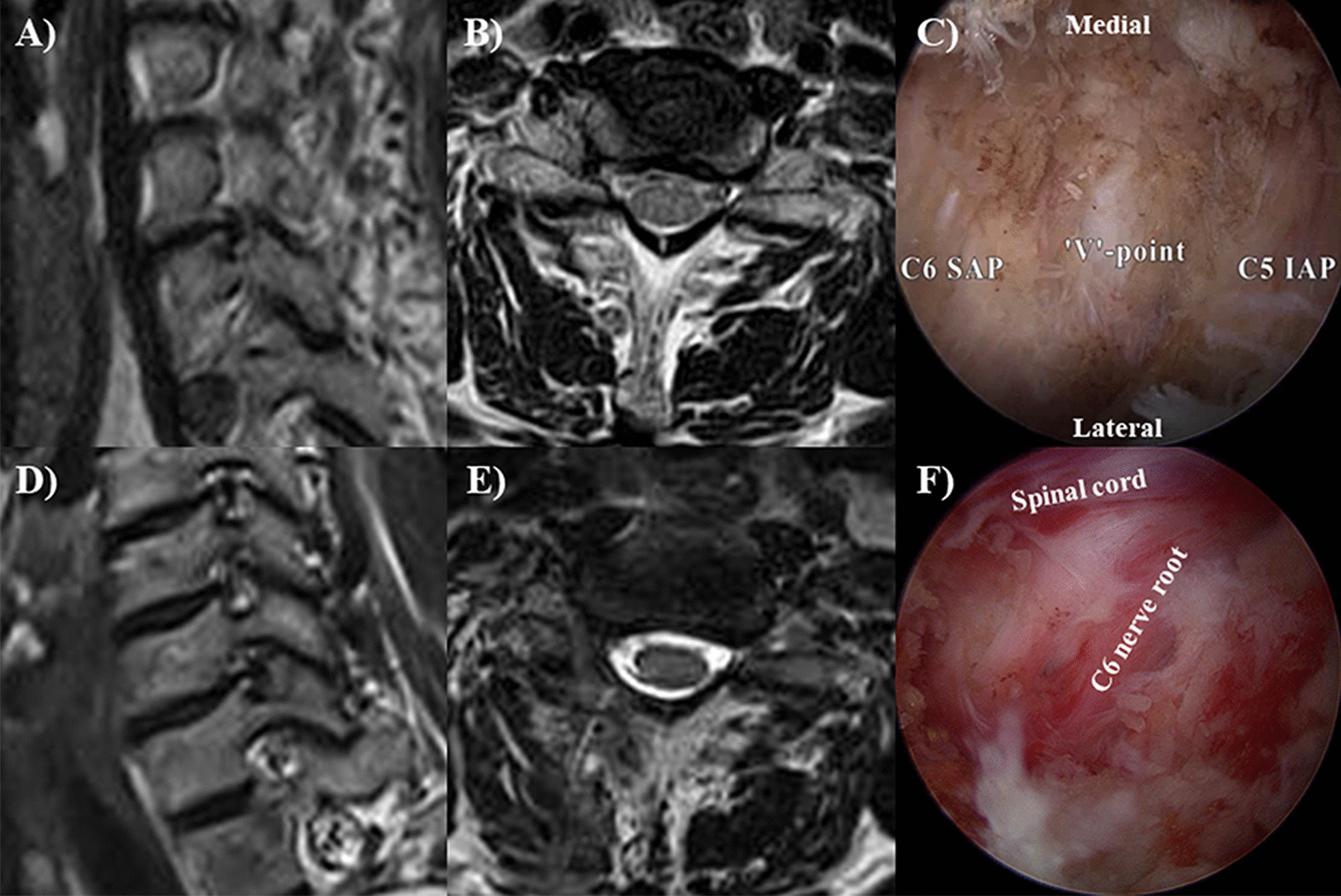
Biportal full-endoscopic posterior cervical foraminotomy that occurred in a 64-year-old woman. The foraminotomy occurred 2 months previously in the 64-year-old woman who had radiating pain and motor weakness in the right upper extremity. T2-weighted **A** right oblique and **B** axial images of pre-operative magnetic resonance imaging (MRI) demonstrating right C5-6 foraminal stenosis; **C** Intraoperative endoscopic image of the “V”-point; **F** After circumferential foraminotomy is performed from the “V”-point, fully decompressed thecal sac and C6 nerve root are found; **D**, **E** Post-operative MRI showing well-decompressed nerve root with minimally damaged paraspinal muscles. The patient’s symptoms also improved

Once the “V”-point and ligamentum flavum located inside were identified, the inferolateral border of the upper lamina, superolateral portion of the lower lamina, and medial portion of the facet joint were drilled out with a 3.5-mm endoscopic drill (Primado II high-speed drill system, NSK, Osaka, Japan) until the attachment of the ligamentum flavum was exposed. After performing circumferential foraminotomy along the pathway of the exiting nerve root, further decompression for the lateral portion of the exiting nerve root with the 1-mm Kerrison punch and small-head curved curettes was performed. Subsequently, piecemeal removal of the lateral extension of the ligamentum flavum commenced. During this process, a vascular complex surrounding the nerve root requires keen attention for possible bleeding, and a curved small bipolar radiofrequency wand (Microblator 30 coblation wand, Smith & Nephew, Tuttlingen, Germany) was useful for vascular coagulation without nerve injury. Sufficient foraminal decompression was ensured by locating the whole exiting nerve root and identifying disc pathology while gently manipulating the exiting nerve root. Using a nerve root retractor, if needed, enough space for discectomy was secured while protecting the spinal cord and exiting nerve root, and limited discectomy was performed (Fig. 2, Additional file 1: Video S1). After meticulous haemostasis, the surgical drain was inserted, and the surgical wound was closed. Post-operatively, all patients were advised to wear a soft neck collar for 2 weeks.

### Data collection

The data collected from the medical records included operative time (i.e. skin incision to closure), length of hospital stay (i.e. from the day of surgery to the day of discharge), and amount of surgical drainage. Moreover, the visual analogue scale (VAS), neck disability score (NDI), and post-operative complications were investigated pre- and post-operatively at 1, 6, and 12 months.

### Statistical analysis

The learning curve cumulative summation analysis (LC-CUSUM) is a modification of CUSUM, which was developed to signal when an adequate level of competence has been achieved from an inadequate level of competence. When a surgeon begins a new surgical procedure, the process is considered “out of control” until the surgeon has reached adequate competency. The LC-CUSUM monitors each subsequent surgery and identifies when the surgical process has come “in control.” Failure was defined as the “inability to complete the process due to a supervisor taking over a technical difficulty, or pain intolerance.” Additionally, an acceptable failure rate was defined as the probability of claiming that a trainee is competent even though they are currently not; in contrast, an unacceptable failure rate was defined as the probability of rejecting the fact that the trainee has reached their competency [9].

The LC was determined by evaluating the operation time using LC-CUSUM in this study. The goal for the operation time was set to 78.5 min, representing the mean operation time (78.50 ± 13.97 min) of PE-PCF performed by a senior neurosurgeon (SY H) who was proficient in OM- and PE-PCF. When the operation time was < 78.5 min, the operation was considered a “procedural success,” whereas “procedural failure” was defined as an operation time of > 78.5 min.

The unacceptable failure rate (ρ^0^), acceptable failure rate (ρ^1^), type I error (α), and type II error (β) should be determined before analysis to make such a control chart. The acceptable and unacceptable failure rates for the “in-control” and “out-of-control” processes were, a priori, set at 20% (ρ^0^ = 0.2) and 40% (ρ^1^ = 0.4), respectively, by a previously published study [10, 11]. The type I (α) and type II (β) error rates were set at 0.05 and 0.20, respectively, and the equations presented in Table 1 were used to calculate the CUSUM score, resulting in a decrease of 0.2933 (S) for each successful operation time and an increase of 0.7067 (1-S) for each failure. A decision limit of h = -2.83 was chosen from the equation, and Excel software was used (Excel 2016; Microsoft, Redmond, Washington, USA) to calculate the LC-CUSUM score.

**Table 1:** Formulas and values involved in plotting the learning curve cumulative summation test

Variable	Value
ρ^0^, unacceptable failure rate	0.4
ρ^1^, acceptable failure rate	0.2
α, probability of the type I error	0.05
β, probability of the type II error	0.2
P = ln(ρ^1^/ ρ^0^)	−0.6932
Q = ln[(1 – ρ^0^)/(1 – ρ^1^)]	−0.2877
S = Q/(P + Q)	0.2933
1-S	0.7067
a = ln[(1 – β)/α]	2.77
h = a/(P + Q), decision limit	−2.83

Descriptive statistics were presented as absolute frequencies for categorical variables and mean with standard deviation (SD) for continuous variables. Dichotomous values were compared using the Chi-squared test, whereas continuous variables were compared between groups using Student’s t test. A *p* value ≤ 0.05 was considered statistically significant. IBM SPSS version 26.0 (SPSS Inc., Chicago, US) was used for data analysis

## Results

This study finally enrolled 50 consecutive patients (36 men; mean age, 52.68 ± 9.56) who underwent single-level BE-PCF. The mean operation time (mOT) was 71.29 ± 11.69 min in BE-PCF, and the detailed demographic data are summarised in Table 2.

**Table 2:** Demographic data and perioperative outcomes

	Early learning period (n = 19)	Late learning period (n = 30)	*p* value
Sex, Male/Female (Male %)	15:4 (78.9)	21:9 (70.0)	0.719
Age (years)	50.63 ± 10.76	54.60 ± 7.78	0.174
Height (cm)	169.63 ± 7.65	167.17 ± 8.59	0.301
Weight (kg)	70.11 ± 8.94	68.63 ± 10.66	0.605
Body mass index (kg/m^2^)	24.32 ± 2.32	24.52 ± 3.07	0.8
ASA score 2 (%)	7 (36.8)	29 (96.7)	< 0.001
Diagnosis (%)			0.216
Contained foraminal HIVD	11 (57.9)	10 (33.3)	
Uncontained foraminal HIVD	4 (21.1)	7 (23.3)	
Foraminal stenosis	4 (21.1)	13 (43.3)	
Level (%)			0.182
C4-5	3 (15.8)	0 (0.0)	
C5-6	6 (31.6)	9 (30.0)	
C6-7	8 (42.1)	16 (53.3)	
C7-T1	2 (10.5)	5 (16.7)	
Side, Right/Left (Right %)	12:7 (63.2)	15:15 (50.0)	0.544
Operation time (min)	71.84 ± 12.61	67.83 ± 10.31	0.254
Length of hospital stay (days)	2.74 ± 1.52	3.20 ± 1.99	0.363
Surgical drainage (ml)	55.53 ± 27.45	40.97 ± 15.57	0.045
Complication (%)	3 (15.8)	1 (3.3)	0.285
Incomplete neural decompression	2 (10.5)	0 (0.0)	
Epidural hematoma	1 (5.3)	1 (3.3)	
Reoperation (%)	1 (5.3)	0 (0.0)	0.388

Data are presented as mean ± SD or as number of patients (percentages)

*ASA* American Society of Anesthesiologist; *HIVD* herniated intervertebral disc

**Fig. 3:**
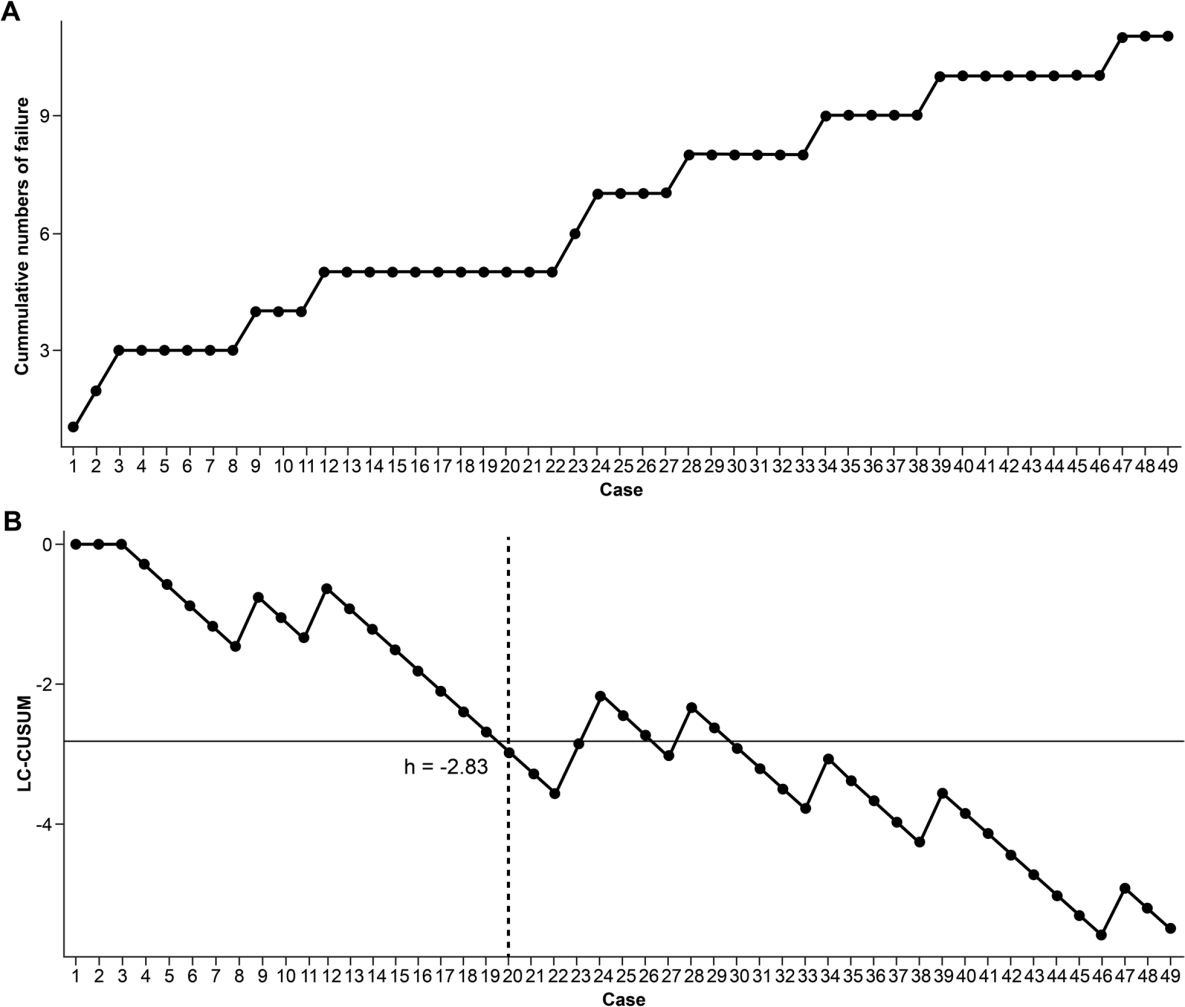
**A** Cumulative numbers of failure; **B** Cumulative summation test for the learning curve signalled competency for surgery at 20 caseloads. Failure is defined as an operation time > 78.5 min. *LC-CUSUM* learning curve cumulative summation test

The cumulative number of failures in BE-PCF is shown in Fig. 3. LC-CUSUM indicated competency for surgery in 20 cases. Therefore, a surgeon with no experience in BE-PCF reached an adequate performance level, corresponding to the mOT of the PE-PCF performed by the senior surgeon in the 20^th^ case.

In BE-PCF, the mOT in the early learning period (< 20 cases) and late learning period (> 20 cases) was 71.84 ± 12.61 and 67.83 ± 10.31 min, respectively (*p* = 0.254). A total of 10 failures were found throughout the total learning period. However, almost all failures (n = 19) were found within the first 20 cases; in the late learning period, only 5 cases exceeded 78.5 min.

The mean length of hospital stay for the first 20 cases was 2.7 ± 1.52 days, which was not significantly different from the late period (3.2 ± 1.99 days; *p* = 0.363). Moreover, the late period had a mean amount of surgical drainage of 40.97 ± 15.57 mL, which was significantly lower than that of the early period (55.53 ± 27.45 Ml, *p* = 0.045).

**Fig. 4:**
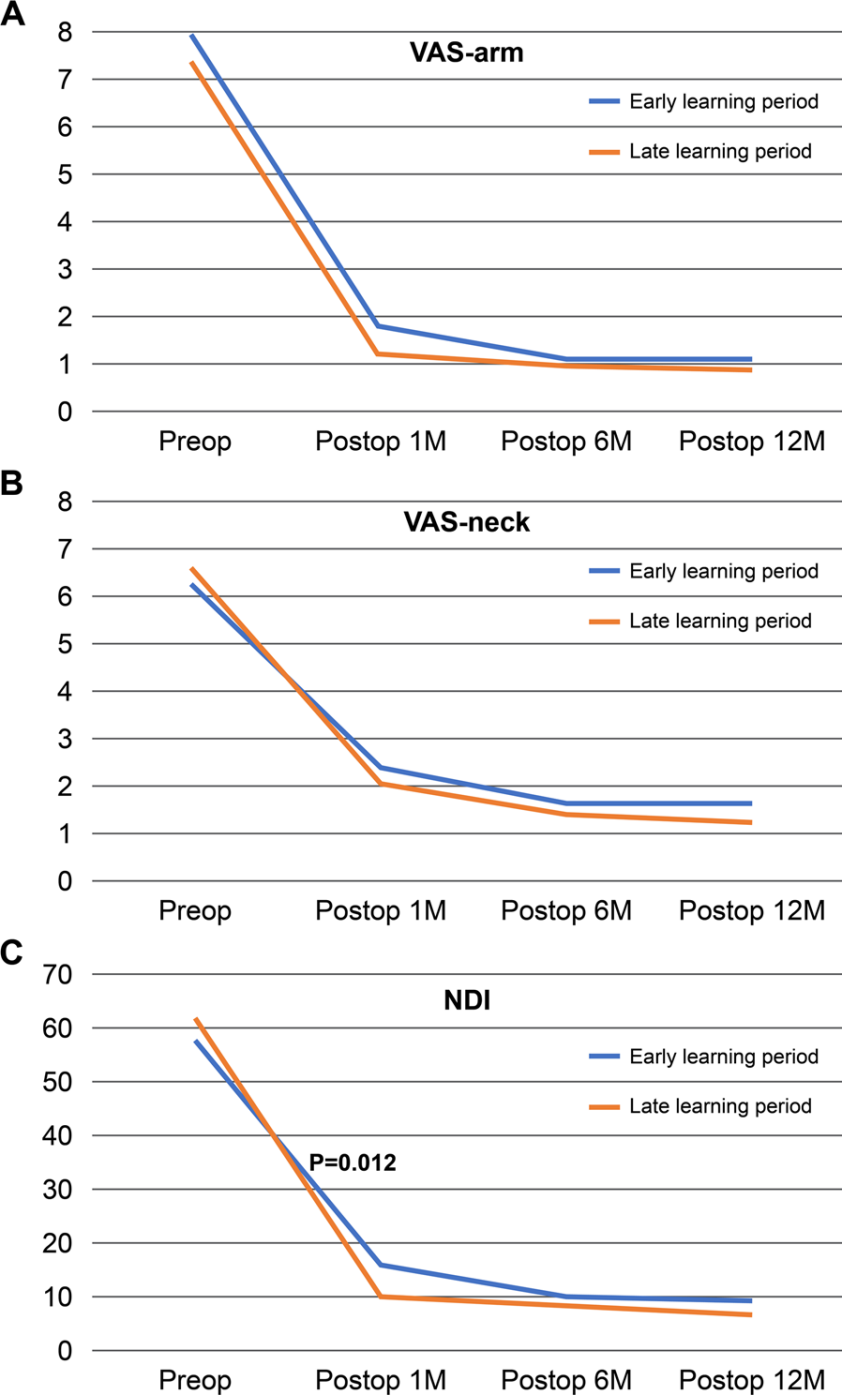
Clinical outcomes between two learning periods **A** VAS-arm, **B** VAS-neck, and **C** NDI. *VAS* visual analogue scale; *NDI* neck disability index

All patients with BE-PCF had significantly improved VAS-arm, VAS-neck, and NDI post-operatively compared to baseline values (*p* < 0.001). There was no statistical difference in clinical outcomes, VAS, and NDI between the early and late periods (*p* > 0.05). However, in the early learning period, NDI at post-operative 1 month (15.68 ± 8.25 vs. 10.13 ± 4.42; *p* = 0.012) was significantly higher (Fig. 4). There were four cases of complications throughout the whole study period, with two cases of incomplete neural decompression and one case of epidural hematoma in the early period and one case of epidural hematoma in the late period (*p* = 0.285). Reoperation was performed in one case due to incomplete neural decompression. And two cases of epidural hematoma were simple hematomas not causing motor weakness, and the pain was improved with conservative treatment (Table 2).

## Discussion

This study used an LC-CUSUM analysis to determine the LC for BE-PCF in the first 50 cases. The LC-CUSUM analysis using mOT signalled that the competency for BE-PCF required 20 cases to reach a senior surgeon’s performance of PE-PCF for the surgeon who was proficient in OM-PCF and had 1 year of experience in biportal endoscopic lumbar surgery. However, when the evaluation was divided into the early and late periods based on 20 cases, there were no significant differences in length of hospital stay, clinical outcomes (VAS and NDI), and post-operative complications.

Since the introduction of percutaneous endoscopic lumbar discectomy (PELD) by Kambin et al. in the mid-1990s [12, 13], endoscopic spine surgery has creatively evolved, with its surgical indication being expanded to the lumbar spine and the cervical and thoracic spines [2]. PE differs from the open microscopic or micro-endoscopic technique in the way the surgeon’s hand works. Generally, in the open microscopic technique, the surgeon works with one hand, whereas the other guides the endoscopic system. However, in PE, the surgeon holds the surgical instruments using the dominant hand and a suction using the non-dominant hand [14]. Moreover, while the direction of the working channel of the full-endoscopy is fixed in-and-out, the microscopic technique does not limit the direction of the work. Notably, it may be difficult for the trainee to adjust the surgical orientation and work directly through an endoscope, and PE is known to be more difficult to learn than microscopy.

However, recently, BE has attracted attention as an alternative to full-endoscopy and microscopic surgery. It is different from PE in that it creates a working space outside the posterior vertebral lamina (or foramen) at the target site and makes two trans-muscular tracts, each at the top and bottom, connected to this space. Moreover, these two tracts are independently used as viewing and working channels. In general, when a right-handed surgeon operates on a patient's left side lesion, the cranial portal is used as the viewing portal and the caudal portal is used as the working portal, and vice versa when operating on the right side lesion. In the BE-PCF, there is no difficulty depending on the location of the lesion because there is no difference in the height of the upper and lower lamina in the target V-point, and this aspect is also one of the advantages of BE. Therefore, BE has the merits of both PE and microscopic surgery, as it allows the surgeon to place an endoscopic lens in the patient’s body just as PE, and it enables the independent use of the surgical instrument with one hand while holding the endoscope with the other just like the microscopic surgery. Therefore, the authors postulated that the LC of both endoscopic surgeries would be different.

LC-CUSUM was developed to determine whether a process has reached a predefined level of performance. It presumes that the process is not in control at the start of monitoring (the trainee is not proficient) and signals when the process can be considered to be in control, that is, when the trainee has reached an acceptable predefined level of performance. In previous studies on PE lumbar discectomy using the LC-CUSUM analysis, 54 caseloads were required to reach appropriate performance [11, 15]. Moreover, the LC of BE lumbar decompressive laminectomy required 58 caseloads to reach adequate performance. Therefore, a substantial learning period comprising approximately 55 cases is required before a spine endoscopist can master IL endoscopic lumbar decompressive laminectomy [10].

Nevertheless, in endoscopic posterior cervical surgery, clinical research, including the LC, has not adequately been studied compared to endoscopic lumbar surgery. Particularly, it is very difficult to determine which technique should be used as a reference for the LC because there is no clear consensus regarding single-level posterior cervical foraminotomy. The clinical outcomes of PE are similar to those of open microscopy; yet, PE may reduce blood loss, length of hospital stays, and post-operative analgesic use. Given this, the LC was determined using a cumulative summation analysis of the mOT of 50 consecutive cases of BE-PCF, with the mOT of PE-PCF performed by a senior surgeon as a control value in this study.

Our study demonstrated that BE-PCF requires a learning period of approximately 20 caseloads to reach the senior surgeon’s performance of PE-PCF in a surgeon who was proficient in OM-PCF and had 1 year of experience with biportal endoscopic lumbar surgery. Moreover, no statistical difference was observed in the length of hospital stay, clinical outcome, and post-operative complications, except for the amount of surgical drain, between the early and late learning periods. However, BE-PCF should not be considered easier to learn than other techniques despite having a shorter learning period than previously reported for IL-PELD and biportal endoscopic lumbar surgery. It is correct to interpret that a substantial learning period is required to overcome the heterogeneity of microscopic and each endoscopic technique and that an additional 20 cases are needed to acquire adequacy in posterior cervical foraminotomy regardless of being already proficient in biportal endoscopic lumbar surgery.

Particularly, to maintain endoscopic visualisation and minimise the risk of neurological complications, there must be an ability to secure an adequate outflow of continuous fluid irrigation in BE [16–19]. However, prolonged operation time, high irrigation pressure, and high irrigation velocity are known risk factors for neurological complications associated with increased intracranial pressure in full-endoscopic spine surgery. Accordingly, BE-PCF may be more advantageous with a significantly shorter mOT than PE-PCF. Comparative studies on the clinical outcomes and safety of posterior cervical foraminotomy using open microscopy, PE, and BE are warranted.

This study has several limitations. First, the study design was retrospective. Second, this study has a relatively small number of cases to determine the LC. Nevertheless, we used an LC-CUSUM analysis, a sequential assessment in which sample sizes are cumulated, to determine the LC of BE-PCF. Third, we analysed only the operation time as a sign of failure or success of the surgery. Although LC can also be determined with other variables, such as the number of radiation exposure, the occurrence of post-operative complications, reoperation, and unsuccessful clinical outcomes, we believe that the surgeon’s competency with the procedure is correlated with a decreased operation time. Lastly, there is a performance bias in this study that as the surgeon has been performing biportal full-endoscopy for the last 1 year before starting BE-PCF. Moreover, the cumulated cases for adequate performance cannot be generalised since the actual LC can vary among individuals.

## Conclusion

In this study, based on the senior surgeon’s performance of PE-PCF, the LC-CUSUM analysis determined that BE-PCF has an LC requiring 20 caseloads to achieve surgical technical competency and significantly reduce the operation time.

## Supplementary Information

**Additional file 1**. Endoscopic visualization of Biportal endoscopic posterior cervical foraminotomy (BE-PCF).

## Data Availability

The datasets used and analysed during the current study available from the corresponding author on reasonable request.

## Abbreviations

BE: Biportal full-endoscopy
IL-PELD: Interlaminar percutaneous endoscopic lumbar discectomy
LC: Learning curve
LC-CUSUM: Learning curve cumulative summation analysis
mOT: Mean operation time
MRI: Magnetic resonance imaging
NDI: Neck disability index
PCF: Posterior cervical foraminotomy
PE: Percutaneous uniportal full-endoscopy
PELD: Percutaneous endoscopic lumbar discectomy
TF-PELD: Transforaminal percutaneous endoscopic lumbar discectomy
VAS: Visual analogue scale
